# Modeling the stochastic within-host dynamics SARS-CoV-2 infection with discrete delay

**DOI:** 10.1007/s12064-022-00379-5

**Published:** 2022-10-03

**Authors:** I. M. Elbaz, M. A. Sohaly, H. El-Metwally

**Affiliations:** 1 Basic Sciences Department, Faculty of Engineering, The British University in Egypt, Cairo, Egypt; 2 Mathematics Department, Faculty of Science, Mansoura University, Mansoura, 35516 Egypt

**Keywords:** Within-host COVID-19 model, Extinction, Persistence, Stochastic perturbation, Delay tactics

## Abstract

In this paper, a new mathematical model that describes the dynamics of the within-host COVID-19 epidemic is formulated. We show the stochastic dynamics of Target-Latent-Infected-Virus free within the human body with discrete delay and noise. Positivity and uniqueness of the solutions are established. Our study shows the extinction and persistence of the disease inside the human body through the stability analysis of the disease-free equilibrium $E_0$ and the endemic equilibrium $E^*$, respectively. Moreover, we show the impact of delay tactics and noise on the extinction of the disease. The most interesting result is even if the deterministic system is inevitably pandemic at a specific point, extinction will become possible in the stochastic version of our model.

## Introduction

The novel coronavirus SARS-CoV-2 is one of the biggest pandemics in history that has been uncovered by the year 2020. The first known infections from SARS-CoV-2 were discovered in Wuhan, Hubei Province, China, in December 2019. The virus affected more than two hundred countries and killed millions of people according to the World Health Organization. The infection can be controlled by physical social distancing, self-isolation at home, face masks, hand-washing and surface cleaning (Lau et al. 2020a). Several countries proposed strict social distancing and lock-down regulations to stop the spread of the virus.

Few research papers could predict the behavior of the COVID-19 disease accurately, and according to WHO, dozens of vaccines candidates are in clinical research and more than ten vaccines are authorized for public use (Vaccine Centre and Medicine 2021; So and Woo 2020). Clinically, there is no effective treatment that can remove the virus from the human body; however, the available treatments help like for Ebola, Influenza and SARS-CoV-1. Several works focused on forecasting the number of infected individuals in populations (El-Metwally et al. 2020; Rahimi et al. 2021; Nabi 2020; Ullah et al. 2019; Elbaz et al. 2022). Forecasting for COVID-19 is very difficult and has failed in many papers because of the type of mathematical models, missing data and/or the random behavior of this virus (Ioannidis et al. 2020). We think it is the time to study the dynamics of the COVID-19 within-host instead of between the human populations.

Many works have dealt with various viruses by mathematical models inside the human body, see Li and Xiao (2011); Zeb et al. (2020); Best and Perelson (2018); Zhang et al. (2020a, 2020b); Zeb et al. (2022). Considering the delay effect in the mathematical modeling of the dynamics of the virus implies right conclusions. It is desirable to propose the within-host COVID-19 model with discrete delay in time. This delay can be embedded in the vaccination process, immune-boosting foods, effective use of antiretroviral therapies, etc.

### Within-host SARS-CoV-2 model

Our proposed model comprises four variable quantities, namely the uninfected pulmonary epithelial targeted cells, *T*(*t*), the latent cells, *L*(*t*) which are infected but not yet infectious, the infected cells, *I*(*t*) and free virus particles, *V*(*t*). Authors in Li et al. (2020b) studied the viral kinetics of COVID-19 without latent class of cells, and we consider the mathematical within-host model in the form

1 $$\begin{aligned} \begin{aligned} {\dot{T}}(t)&= d_1 T(0) - \beta e^{-d_{3} \tau } T(t-\tau ) V(t-\tau ) - d_1 T(t), \\ {\dot{L}}(t)&= \beta e^{-d_{3} \tau } T(t-\tau ) V(t-\tau ) - (d_2 + k) L(t), \\ {\dot{I}}(t)&= k L(t) - d_3 I(t), \\ {\dot{V}}(t)&= p I(t) - d_4 V(t). \end{aligned} \end{aligned}$$

For $\theta \in \left[ -\tau , 0 \right]$, the initial conditions of this model are

2 $$\begin{aligned} T(\theta )=\, & {} \phi _1(\theta ), \, \, L(\theta ) = \phi _2(\theta ), \, \, I(\theta ) = \phi _3(\theta ), \, \, \nonumber \\&V(\theta ) = \phi _4(\theta ), \quad \phi _i(\theta ) \ge 0 \, \forall \, i = 1,\cdots ,4. \end{aligned}$$

**Fig. 1:**
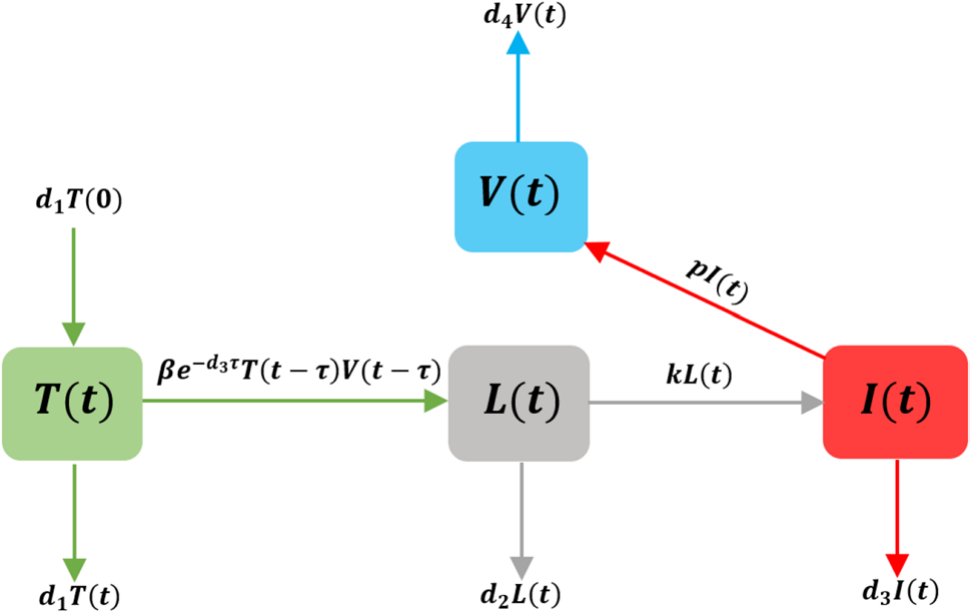
Flow map of the delayed COVID-19 model within-host (1)

Figure 1 shows the flux of this model. Solutions of (1) with (2) are in ${\mathcal {C}}^4$, and still nonnegative, where ${\mathcal {C}}: \left[ -\tau ,0 \right] \rightarrow {\mathbb {R}}_+$ is a Banach space of all continuous functions with the norm

$$\begin{aligned} \Vert \phi \Vert = \sup _{\theta \in \left[ -\tau ,0 \right] } \vert \phi (\theta ) \vert . \end{aligned}$$

The model assumes that there is a constant of regeneration $d_1 T(0)$ susceptible target cells. The susceptible target cells are infected by free virus particles with a bilinear incidence rate $\beta TV$, and these infected cells produce with a rate *p* free virus particles. Parameters $d_1, \, d_2, \, d_3,$ and $d_4$ are the death rates of the susceptible target cells, latent cells, infected cells and free virus particles, respectively. Latent cells on an average span 1/*k* units of time in *L* class and then join the infected class of cells. It should be noted that $d_1$ is a natural death rate or natural clearance rate while $d_2, \, d_3$ and $d_4$ are a combination of the natural clearance rate and the role of immune system in the elimination of these cells. The superior limit of the time delay is $\tau$. The probability of surviving from $t-\tau$ to *t* is $e^{-d_{3} \tau }$, and then $\beta e^{-d_{3} \tau } T(t-\tau ) V(t-\tau )$ is the force of infection rate with discrete delay.

### Basic reproduction number and equilibrium states

The three infected compartments are

$$\begin{aligned} \dfrac{\mathrm{d}}{\mathrm{d}t} \left[ \begin{array}{c} L(t) \\ I(t) \\ V(t) \end{array} \right] = {\mathsf {F}}-{\mathsf {V}}, \end{aligned}$$

where

$$\begin{aligned} {\mathsf {F}} = \left[ \begin{array}{c} \beta e^{-d_{3} \tau } T(t-\tau ) V(t-\tau ) \\ 0 \\ 0 \end{array} \right] , \, \, \, \, {\mathsf {V}} = \left[ \begin{array}{c} (d_2 + k) L(t) \\ d_3 I(t) - k L(t) \\ d_4 V(t) - p I(t) \end{array} \right] . \end{aligned}$$

The Jacobians of ${\mathsf {F}}$ and ${\mathsf {V}}$ are

$$\begin{aligned} {\mathsf {F}}_0 = \left[ \begin{array}{ccc} 0{\quad } &{} 0{\quad } &{} \beta e^{-d_{3} \tau } T_0 \\ 0{\quad } &{} 0{\quad } &{} 0 \\ 0{\quad } &{} 0{\quad } &{} 0 \end{array} \right] , \quad {\mathsf {V}}_0 = \left[ \begin{array}{ccc} d_2 + k {\quad } &{} 0{\quad } &{} 0 \\ -k{\quad } &{} d_3{\quad } &{} 0 \\ 0 {\quad } &{} - p {\quad } &{} d_4 \end{array} \right] . \end{aligned}$$

The Next Generation Matrix is

$$\begin{aligned} {\mathsf {F}}_0 {\mathsf {V}}_0^{-1} = \left[ \begin{array}{ccc} \dfrac{\beta e^{-d_{3} \tau } T_0 k p}{(d_2 + k) d_3 d_4} &{} \dfrac{\beta e^{-d_{3} \tau } T_0 p}{d_3 d_4} &{} \dfrac{\beta e^{-d_{3} \tau } T_0}{d_4} \\ 0 &{} 0 &{} 0 \\ 0 &{} 0 &{} 0 \end{array} \right] , \end{aligned}$$

with eigenvalues

$$\begin{aligned} \left[ \begin{array}{c} 0 \\ 0 \\ \dfrac{\beta e^{-d_{3} \tau } T_0 k p}{(d_2 + k) d_3 d_4} \end{array} \right] \end{aligned}$$

The basic reproduction number $R_0^d$ is the spectral radius of ${\mathsf {F}}_0 {\mathsf {V}}_0^{-1}$ or its maximum eigenvalue, then

$$\begin{aligned} R_0^d = \frac{\beta e^{-d_{3} \tau } T(0) k p}{(d_2 + k) d_3 d_4}. \end{aligned}$$

The disease dies out and the number of free virus particles goes to zero for $R_0^d < 1$, and the disease persists for $R_0^d > 1$. Clearly, we have two equilibrium states at most, $E_0 = (T_0,L_0,I_0,V_0) = (T(0),0,0,0)$ is the infection-free equilibrium state, and a positive endemic equilibrium state

$$\begin{aligned} E^*= \,& {} (T^*,L^*,I^*,V^*)= \left( \frac{(d_2 + k)d_3 d_4}{\beta k p}, \frac{d_1 T(0)}{d_2 + k} - \frac{d_1 d_3 d_4}{\beta k p}, \right. \\&\left. \frac{d_1 k T(0)}{d_3 (d_2 + k)} - \frac{d_3 d_4}{\beta p}, \frac{d_1 T(0) k p}{d_3 d_4 (d_2 + k)} - \frac{d_1}{\beta } \right) . \end{aligned}$$

System (1) is exposed to some stochastic parametric perturbations in the form of environmental noises. We have to consider such models for best control and to capture all possible types of uncertainty. Many authors have proposed stochastic models in many disciplines in El-Metwally et al. (2021); Zhang and Alzahrani (2020); Tesfay et al. (2021) and with discrete delay in El-Metwally et al. (2021); Almutairi et al. (2021).

Define $J := C([-\tau ,0],L_2)$, a Banach space of mean-square continuous functionals $\varphi$ defined on $[-\tau ,0]$ with the norm

$$\begin{aligned} \Vert \varphi \Vert _J = \sup _{-\tau \le s \le 0} \Vert \varphi \Vert _2 = \sup _{-\tau \le s \le 0} \left( {\mathbb {E}} \left[ \varphi ^2(s) \right] \right) ^{1/2}. \end{aligned}$$

Many works related to the mean-square sense can be found in Yassen et al. (2016, 2017); Sohaly et al. (2018); Elbaz (2021). Unless otherwise stated, let $(\Omega ,{\mathcal {F}},\left\{ {\mathcal {F}}_{t}\right\} _{t \ge 0},{\mathbb {P}})$ be a complete filtered probability space satisfies the condition of the right continuity and ${\mathcal {F}}_0$ contains ${\mathbb {P}}$-null sets. We introduce the stochastic version of the delayed within-host COVID-19 epidemic in the form

3 $$\begin{aligned} \begin{aligned}&\mathrm{d}T(t) = \left( d_1 T(0) - \beta e^{-d_{3} \tau } T(t-\tau ) V(t-\tau ) - d_1 T(t)\right) \mathrm{d}t \\&\qquad - \sigma T(t-\tau ) V(t-\tau ) dB(t), \\&\mathrm{d}L(t) = \left( \beta e^{-d_{3} \tau } T(t-\tau ) V(t-\tau ) - (d_2 + k) L(t)\right) \mathrm{d}t \\&\qquad + \sigma T(t-\tau ) V(t-\tau ) \mathrm{d}B(t), \\&\mathrm{d}I(t) = \left( k L(t) - d_3 I(t)\right) \mathrm{d}t, \\&\mathrm{d}V(t) = \left( p I(t) - d_4 V(t)\right) \mathrm{d}t. \end{aligned} \end{aligned}$$

The stochastic process *B*(*t*) is a one-dimensional standard Wiener process defined on the complete filtered probability space $(\Omega ,{\mathcal {F}},{\mathcal {F}}_{t}^{B},{\mathbb {P}})$, where ${\mathcal {F}}_{t}^{B}$ is the filtration generated by it up to time *t*. Set

$$\begin{aligned} R_0^s = \frac{\beta e^{-d_{3} \tau } T(0) k p }{(d_2 + k) d_3 d_4 + \frac{1}{2} \sigma ^2 T_0^2}, \end{aligned}$$

as the basic reproduction number of this stochastic system.

Well-posedness of this system is shown in the next section. Extinction and persistence of the virus within the human body are shown in Sect. 3. Stability areas and some computer simulations are carried out in Sect. 4. At the end of the paper, we state our conclusions.

## Well-posedness of (3)

This section is devoted to prove that for any given initial value, the solution is nonnegative and global, i.e., no explosion in a finite time. The coefficients of (3) are required to satisfy the local Lipschitz condition and the condition of linear growth (Mao 2007). Anyway, the coefficients of (3) are only satisfy the local Lipschitz condition; consequently, the solution may explode in finite time. By introducing an appropriate Lyapunov function, we show that the solution is nonnegative and global.

### Lemma 2.1

A unique global solution $(T(t),L(t),I(t),V(t)) \in {\mathbb {R}}_{+}^{4}$ of (3) exists for all $t \ge 0$ for any initial state

4 $$\begin{aligned} (T(\theta ),L(\theta ),I(\theta ),V(\theta )) = (\phi _1(\theta ),\phi _2(\theta ),\phi _3(\theta ),\phi _4(\theta )) \in {\mathbb {R}}_{+}^{4}. \end{aligned}$$

Moreover, it is bounded and remains in ${\mathbb {R}}_{+}^{4}$ almost surely.

### Proof

In (3), the drift and the diffusion terms are mean-square locally Lipschitz as for positive constant *K* and $g(t,x_t) : [0,T] \times L_2(\Omega ) \rightarrow L_2(\Omega )$,

$$\begin{aligned} \Vert g_i(t,x_t) - g_i(t,y_t) \Vert _2 \le K \Vert x_t - y_t\Vert _2, \quad i = 1,2. \end{aligned}$$

for $g_1(t,x_t) = -\sigma T(t-\tau )V(t-\tau )$, and $g_2(t,x_t) = \sigma T(t-\tau )V(t-\tau )$. The coefficients in (3) are continuous functionals and by taking the delay into account, we can assume that the drift and the diffusion terms satisfy for arbitrary continuous functions $\psi , \xi \in {\mathcal {C}}[-\tau ,0]$

$$\begin{aligned} \begin{aligned} |f_i(t,\psi ) - f_i(t,\xi )|^2&\le \int _{-\tau }^{0} |\psi (s) - \xi (s)|^2 \mathrm{d}K_1(s), \quad i = 1,\cdots ,4. \\ |g_i(t,\psi ) - g_i(t,\xi )|^2&\le \int _{-\tau }^{0} |\psi (s) - \xi (s)|^2 \mathrm{d}K_2(s), \quad i = 1,2. \end{aligned} \end{aligned}$$

for nondecreasing bounded functions $K_1,K_2$ and

$$\begin{aligned} \begin{aligned} f_1&= d_1 T(0) - \beta e^{-d_{3} \tau } T(t-\tau ) V(t-\tau ) - d_1 T(t), \\ f_2&= \beta e^{-d_{3} \tau } T(t-\tau ) V(t-\tau ) - (d_2 + k) L(t), \\ f_3&= k L(t) - d_3 I(t), \quad f_4 = p I(t) - d_4 V(t). \end{aligned} \end{aligned}$$

Then for any initial condition (4) such that

$$\begin{aligned} \sup _{-\tau \le s \le 0} {\mathbb {E}} |\phi (s)|^2 = \sup _{-\tau \le s \le 0} \Vert \phi (s) \Vert _2^2 < \infty , \end{aligned}$$

the system (3) admits a maximal unique local solution $(T(t),L(t),I(t),V(t)) \in [-\tau ,\tau _1]$ where $\tau _1$ is the explosion time. If $\tau _1 = \infty$, then the solution is global. Assume that every $\phi _i(\theta ) \in {\mathbb {R}}_{+}^{4}, \, \theta \in [-\tau ,0], i = 1, \cdots , 4$, lies within the interval $\left[ \dfrac{1}{k_1},k_1 \right] , \, k_1 > 0$.

Define the stopping time

$$\begin{aligned} \tau _k = \inf \left\{ t \in \left[ 0, \tau _1 \right] : (T(t),L(t),I(t),V(t)) \ne \left[ \dfrac{1}{k},k \right] \right\} , \end{aligned}$$

this stopping time $\tau _k$ increases as $k \rightarrow \infty$. Set $\tau _\infty = \lim _{k \rightarrow \infty } \tau _k$ whence $\tau _\infty \le \tau _1$ a.s.

Assume $\tau _k < \infty ,$ i.e., $\tau _\infty = \infty$ is not satisfied, then

5 $$\begin{aligned} {\mathbb {P}}(\tau _{\infty } \le T )> \varepsilon , \, \, \text {for} \, \, T > 0, 0< \varepsilon < 1. \end{aligned}$$

So, there exists $k \ge k_1$ such that

6 $$\begin{aligned} {\mathbb {P}}(\tau _{k} < T )> 0, \, \, \text {for} \, \, T >0. \end{aligned}$$

Consider the Lyapunov functional in the form

$$\begin{aligned} W= \,& {} (T(t) - 1 - \ln T(t)) + (L(t) - 1 - \ln L(t)) + (I(t) - 1 \\&- \ln I(t)) + (V(t) - 1 - \ln V(t)). \end{aligned}$$

Using the Itô formula of the stochastic integral (Almutairi et al. 2021), we get

$$\begin{aligned} \begin{aligned} \mathrm{d}W =&\left( 1 - \dfrac{1}{T(t)} \right) \mathrm{d}T(t) + \dfrac{1}{2T^{2}(t)}(\mathrm{d}T(t))^{2} \\&+ \left( 1 - \dfrac{1}{L(t)} \right) \mathrm{d}L(t) + \dfrac{1}{2L^{2}(t)}(\mathrm{d}L(t))^{2} \\&+ \left( 1 - \dfrac{1}{I(t)} \right) \mathrm{d}I(t) + \dfrac{1}{2I^{2}(t)}(\mathrm{d}I(t))^{2} \\&+ \left( 1 - \dfrac{1}{V(t)} \right) \mathrm{d}V(t) + \dfrac{1}{2V^{2}(t)}(\mathrm{d}V(t))^{2} \\ =&\left( 1 - \dfrac{1}{T(t)} \right) \left( \left( d_1 T(0) - \beta e^{-d_{3} \tau } T(t-\tau ) V(t-\tau ) \right. \right. \\&\left. \left. - d_1 T(t) \right) dt - \sigma T(t-\tau ) V(t-\tau ) \mathrm{d}B(t) \right) \\&+ \left( 1 - \dfrac{1}{L(t)} \right) \left( \left( \beta e^{-d_{3} \tau } T(t-\tau ) V(t-\tau ) \right. \right. \\&\left. \left. - (d_2 + k) L(t)\right) \mathrm{d}t + \sigma T(t-\tau ) V(t-\tau ) \mathrm{d}B(t) \right) \\&+ \left( 1 - \dfrac{1}{I(t)} \right) \left( k L(t) - d_3 I(t) \right) \mathrm{d}t \\&+ \left( 1 - \dfrac{1}{V(t)} \right) \left( p I(t) - d_4 V(t) \right) \mathrm{d}t \\&+ \dfrac{1}{2L^{2}(t)} \sigma ^2 T^2(t-\tau ) V^2(t-\tau ) \mathrm{d}t \\&+ \dfrac{1}{2T^{2}(t)} \sigma ^2 T^2(t-\tau ) V^2(t-\tau ) \mathrm{d}t \\ =&\left( d_1 T(0) - d_1 T(t) - \frac{d_1 T(0)}{T(t)} + \beta e^{-d_{3} \tau } T(t-\tau )\right. \\&V(t-\tau ) \left( \frac{1}{T(t)} - \frac{1}{L(t)}\right) - k \frac{L(t)}{I(t)} \\&+ d_1 + d_2 + d_3 + d_4 + k -d_2 L(t) - d_3 I(t) \\&- d_4 V(t) + pI(t) - p \frac{I(t)}{V(t)} \\&\left. + \frac{1}{2} \sigma ^2 T^2(t-\tau ) V^2(t-\tau ) \left( \frac{1}{T^2(t)} + \frac{1}{L^2(t)}\right) \right) \mathrm{d}t \\&+ \sigma T(t-\tau ) V(t-\tau )\left( \frac{1}{T(t)} - \frac{1}{L(t)}\right) \mathrm{d}B(t). \end{aligned} \end{aligned}$$

Then

$$\begin{aligned} \begin{aligned} W&(t,T(t),L(t),I(t),V(t))- W(t,\phi _1(0),\phi _2(0),\phi _3(0),\phi _4(0)) \\&\le \int _{0}^{t} \left( d_1 T(0) + \frac{\beta e^{-d_{3} \tau } T(s-\tau ) V(s-\tau )}{T(s)} \right. \\&\quad + k + p I(s) + \sum _{i=1}^{4} d_i \\&\quad \left. + \frac{1}{2} \sigma ^2 T^2(s-\tau ) V^2(s-\tau ) \left( \frac{1}{T^2(s)} + \frac{1}{L^2(s)}\right) \right) \mathrm{d}s \\&\quad + \sigma \int _{0}^{t} T(s-\tau ) V(s-\tau )\left( \frac{1}{T(s)} - \frac{1}{L(s)}\right) \mathrm{d}B(s). \end{aligned} \end{aligned}$$

Taking the expectation leads to

$$\begin{aligned} \begin{aligned} {\mathbb {E}}&\left[ W(t,T(t \wedge \tau _k),L(t \wedge \tau _k),I(t \wedge \tau _k),V(t \wedge \tau _k)) \right] \\&\quad - {\mathbb {E}} \left[ W(t,\phi _1(0),\phi _2(0),\phi _3(0),\phi _4(0))\right] \\&\le {\mathbb {E}} \left[ \int _{0}^{t \wedge \tau _k} \left( d_1 T(0) + \sum _{i=1}^{4} d_i \right. \right. \\&\quad \left. \left. + k + (p + \beta ) M + \frac{1}{2} \sigma ^2 M^2 \right) \mathrm{d}s \right] := K, \end{aligned} \end{aligned}$$

where *K* is a suitable constant independent of *T*, *L*, *I*, *V*. Then

$$\begin{aligned} {\mathbb {E}}\left[ W(T,L,I,V)\right] \Big |_{t \wedge \tau _{k}} \le K. \end{aligned}$$

Set $\Omega = \left\{ \tau _{k} \le T \right\} \, \forall \, k \ge k_{1}$. And ${\mathbb {P}}(\Omega _{k}) > \varepsilon$ by (6). At least one of $T(\tau _{k},\omega ),L(\tau _{k},\omega ),I(\tau _{k},\omega ),V(\tau _{k},\omega )$ equals *k* or 1/*k* where $\omega \in \Omega _k$. Hence, from (5), we have

$$\begin{aligned} K\ge & {} {\mathbb {E}} \left[ 1_{\Omega _{k}(\omega )} W\left( T(\tau _{k},\omega ),L(\tau _{k},\omega ),I(\tau _{k},\omega ),V(\tau _{k},\omega )\right) \right] \\\ge & {} \varepsilon \left( (k-\ln k) \wedge (\dfrac{1}{k}-\ln \dfrac{1}{k}) \right) , \end{aligned}$$

where $1_{\Omega _{k}}$ is the indicator function of $\Omega _{k}(\omega )$. Let $k \rightarrow \infty$, then $\infty > K = \infty$, a contradiction arises here. So $\tau _{\infty } = \infty$ a.s.

Regarding the boundedness of the solutions of (3), the total population of cells $N(t) = T(t) + L(t) + I(t)$, where

$$\begin{aligned} \begin{aligned} \dfrac{\mathrm{d}N(t)}{\mathrm{d}t}&= d_1 T(0) - d_1 T(t) - d_2 L(t) - d_3 I(t) \\&\le d_1 T(0) - \min \lbrace d_1, d_2, d_3\rbrace \left( T(t) + L(t) + I(t) \right) . \end{aligned} \end{aligned}$$

Assume that $d = \min \lbrace d_1, d_2, d_3\rbrace$, then

$$\begin{aligned} \dfrac{\mathrm{d} {\mathbb {E}}\left[ N(t)\right] }{\mathrm{d}t} \le d_1 T(0) - \mathrm{d} {\mathbb {E}}\left[ N(t)\right] , \end{aligned}$$

and

$$\begin{aligned} \limsup _{t \rightarrow \infty } {\mathbb {E}}\left[ N(t)\right] \le \frac{d_1 T(0)}{d}. \end{aligned}$$

Consequently, all solutions of (3) with respect to the initial conditions (4) are bounded in a biologically feasible region

$$\begin{aligned} \Gamma = \left\{ \left( T(t), L(t), I(t) \right) \in {\mathbb {R}}_+^3 \Big | T(t) + L(t) + I(t) \le \frac{d_1 T(0)}{d} \right\} . \end{aligned}$$

Clearly, the number of free virus particles is also bounded at any time *t*. $\square$

## Extinction and persistence

In this section, we seek for the sufficient conditions for stochastic stability (stability in probability) of the equilibrium states of (3). This can be done by investigating the necessary conditions for the mean-square stability of the zero solution of the corresponding linear system which are in the same time sufficient for stability in probability of the equilibrium state of the nonlinear system. We begin with centering the nonlinear system around the equilibrium point and linearizing.

### **Stability of the disease-free equilibrium**

By centering the system (3) around $E_0$ using the transformations

7 $$\begin{aligned}&T = x_1 + T(0), \quad L = x_2, \quad I = x_3, \quad \text {and} \quad V = x_4. \nonumber \\&\begin{aligned} \mathrm{d}x_1(t) =&\left( d_1 T(0) - \beta e^{-d_{3} \tau } (x_1(t-\tau )\right. \\&\left. +T(0)) x_4(t-\tau ) - d_1 (x_1(t) + T(0)) \right) \mathrm{d}t \\&- \sigma (x_1(t-\tau )+T(0)) x_4(t-\tau ) \mathrm{d}B(t), \\ \mathrm{d}x_2(t) =&\left( \beta e^{-d_{3} \tau } (x_1(t-\tau )+T(0)) x_4(t-\tau )\right. \\&\left. - (d_2 + k) x_2(t)\right) \mathrm{d}t \\&+ \sigma (x_1(t-\tau )+T(0)) x_4(t-\tau ) \mathrm{d}B(t), \\ \mathrm{d}x_3(t) =&\left( k x_2(t) - d_3 x_3(t)\right) \mathrm{d}t, \\ \mathrm{d}x_4(t) =&\left( p x_3(t) - d_4 x_4(t) \right) . \end{aligned} \end{aligned}$$

The corresponding linear system is

8 $$\begin{aligned} \begin{aligned} \mathrm{d}x_1(t) =&\left( - \beta e^{-d_{3} \tau } x_4(t-\tau ) - d_1 x_1(t) \right) \mathrm{d}t \\&- \sigma T(0) x_4(t-\tau ) \mathrm{d}B(t), \\ \mathrm{d}x_2(t) =&\left( \beta e^{-d_{3} \tau } x_4(t-\tau ) - (d_2 + k) x_2(t) \right) \mathrm{d}t \\&+ \sigma T(0) x_4(t-\tau ) \mathrm{d}B(t), \\ \mathrm{d}x_3(t) =&\left( k x_2(t) - d_3 x_3(t) \right) \mathrm{d}t, \\ \mathrm{d}x_4(t) =&\left( p x_3(t) - d_4 x_4(t) \right) \mathrm{d}t. \end{aligned} \end{aligned}$$

#### Lemma 3.1

The zero solution of (8) is stable in mean-square if $R_0^s < 1$ and

9 $$\begin{aligned} \beta e^{-d_3 \tau }< \min \left\{ 2 d_1, 2(d_2 + k) \right\} , \quad k< 2 d_3, \quad p < 2d_4. \end{aligned}$$

#### Proof

Choose the Lyapunov functional $W = W_1 + W_2$, where $W_1(t,x_t) = x_1^2(t) + Ax_2^2(t) + Bx_3^2(t) + Cx_4^2(t),$ and *A*, *B*, *C* are arbitrary positive quantities to be determined. Then

$$\begin{aligned} \begin{aligned}&\mathrm {L}W_1(t,x_t) = 2x_1(t) (- \beta e^{-d_{3} \tau } x_4(t-\tau ) - d_1 x_1(t)) \\&\qquad + 2 A x_2(t) (\beta e^{-d_{3} \tau } x_4(t-\tau ) - (d_2 + k) x_2(t)) \\&\qquad + \sigma ^2 (A+1) T^2_0 x_4^2(t-\tau ) + 2 B x_3(k x_2(t) - d_3 x_3(t)) \\&\qquad + 2 C x_4(p x_3(t) - d_4 x_4(t))\\&\quad \le \beta T_0 e^{-d_{3} \tau } \left( x_1^2(t) + x_4^2(t-\tau ) \right) - 2d_1 x_1^2(t) \\&\qquad + \sigma ^2 (A+1) T^2_0 x_4^2(t-\tau ) \\&\qquad + \beta T_0 A e^{-d_{3} \tau } \left( x_2^2(t) + x_4^2(t-\tau ) \right) - 2 A (d_2 + k) x_2^2(t) \\&\qquad + B k \left( x_3^2(t) + x_2^2(t) \right) \\&\qquad - 2 B d_3 x_3^2(t) + C p \left( x_4^2(t) + x_3^2(t) \right) - 2 d_4 C x_4^2(t) \\&\quad = \left( \beta T_0 e^{-d_{3} \tau } - 2d_1 \right) x_1^2(t) + \left( \beta T_0 A e^{-d_{3} \tau } + B k \right. \\&\qquad \left. - 2A(d_2 + k) \right) x_2^2(t) \\&\qquad + \left( (k-2d_3)B + C p \right) x_3^2(t) + \left( (p-2d_4)C \right) x_4^2(t) \\&\qquad + (A+1) \left( \beta T_0 e^{-d_{3} \tau } + \sigma ^2 T^2_0 \right) x_4^2(t-\tau ). \end{aligned} \end{aligned}$$

For the negative definiteness of $\mathrm {L}W$ along the trajectory of the solution, we choose the second component of *W* to be

$$\begin{aligned} W_2 = \left( \beta T_0 e^{-d_{3} \tau } + \sigma ^2 T^2_0 \right) (A+1) \int _{t-\tau }^{t} x^2_4(s) \mathrm{d}s. \end{aligned}$$

Then

10 $$\begin{aligned} \begin{aligned}&\mathrm {L}W(t,x_t) \le \left( \beta T_0 e^{-d_{3} \tau } - 2d_1 \right) x_1^2(t) \\&\qquad + \left( \left( \beta T_0 e^{-d_{3} \tau } - 2(d_2 + k) \right) + B k \right) x_2^2(t). \\&\qquad + \left( (k-2d_3)B + C p \right) x_3^2(t) + \left( (p-2d_4)C\right. \\&\qquad \left. + \left( \beta T_0 e^{-d_{3} \tau } + \sigma ^2 T^2_0 \right) (A+1) \right) x_4^2(t). \end{aligned} \end{aligned}$$

Using (9), choose

$$\begin{aligned} \left\{ \begin{array}{cc} \begin{aligned} &{}A = \frac{-2\beta k}{\beta T_0 e^{-d_{3} \tau } - 2(d_2 + k) \mu },\\ &{}B = \frac{-2 C p}{k - 2 d_3},\\ &{}C = \frac{-\left( \beta T_0 e^{-d_{3} \tau } + 2 \sigma ^2 T^2_0 \right) (A+1)}{p - 2 d_4}.\\ \end{aligned} \end{array}\right. \end{aligned}$$

Consequently,

$$\begin{aligned} \begin{aligned} {\mathbb {E}} \left[ \mathrm {L}W(t,x_t)\right]&\le \left( \beta T_0 e^{-d_{3} \tau } - 2d_1 \right) {\mathbb {E}} |x_1(t)|^2 - B k {\mathbb {E}} |x_2(t)|^2 - \sigma ^2 T^2_0 {\mathbb {E}} |x_3(t)|^2 \\&\le - \min \left\{ 2d_1 - \beta T_0 e^{-d_{3} \tau }, B k, \sigma ^2 T^2_0 \right\} {\mathbb {E}} |x(t)|^2, \end{aligned} \end{aligned}$$

hence the zero solution of (8) is mean-square stable. $\square$

#### Theorem 3.1

Conditions (9) are sufficient for stability in probability of the disease-free equilibrium $E_0$ of the nonlinear system (3) or the trivial equilibrium of (7).

#### Proof

Following the same argument of the previous lemma by choosing $W_1(t,x_t)$ in the form

$$\begin{aligned} W_1(t,x_t) = D T(0) x_1^2(t) + E T(0) x_2^2(t) + F x_3^2(t) + G x_4^2(t), \end{aligned}$$

where *D*, *E*, *F*, *G* are positive quantities to be determined. Then according to (7)

$$\begin{aligned} \begin{aligned}&\mathrm {L}W_1(t,x_t) = 2DT(0)x_1(t) \left( - \beta e^{-d_{3} \tau } (x_1(t-\tau )\right. \\&\qquad \left. +T(0))x_4(t-\tau ) - d_1 x_1(t)\right) \\&\qquad + T(0) \sigma ^2 \left( x_1(t-\tau )+T(0)\right) ^2 x_4^2(t-\tau )(D + E) \\&\qquad + 2ET(0)x_2(t) \left( - \beta e^{-d_{3} \tau } (x_1(t-\tau )+T(0))x_4(t-\tau )\right. \\&\qquad \left. - (d_2 + k) x_2(t)\right) \\&\qquad + 2Fx_3(t) \left( k x_2(t) - d_3 x_3(t)\right) \\&\qquad + 2Gx_4(t) \left( p x_3(t) - d_4 x_4(t)\right) \\&\quad \le \beta e^{-d_{3} \tau } D T(0) \left( x_1^2(t) + (x_1(t-\tau )\right. \\&\qquad \left. +T(0))^2 x_4^2(t-\tau ) \right) - 2d_1 D x_1^2(t) \\&\qquad + T(0) (D + E) \sigma ^2 \left( x_1(t-\tau )+T(0)\right) ^2 x_4^2(t-\tau ) \\&\qquad -2 E (d_2 + k) x_2^2(t)\\&\qquad + \beta e^{-d_{3} \tau } E T(0) \left( x_2^2(t) + (x_1(t-\tau )+T(0))^2 x_4^2(t-\tau ) \right) \\&\qquad + F k \left( x_2^2(t) + x_3^2(t)\right) \\&\qquad - 2 d_3 F x_3^2(t) + G p \left( x_3^2(t) + x_4^2(t)\right) - 2 d_4 G x_4^2(t)\\&\quad = \left( \beta T(0) e^{-d_3 \tau } \right) D x_1^2(t) + \left( \left[ \beta T(0) e^{-d_3 \tau } \right. \right. \\&\qquad \left. \left. - 2(d_2 + k)\right] E + F k \right) x_2^2(t) \\&\qquad + \left( (k-2d_3) F + G p \right) x_3^2(t) + \left( (p-2d_4) G\right) x_4^2(t) \\&\qquad + \beta T(0) e^{-d_3 \tau }(\delta + T(0))^2 (D+E) x_4^2(t-\tau ). \end{aligned} \end{aligned}$$

Now, we can choose

$$\begin{aligned} W_2(t,x_t) = \beta T(0) e^{-d_3 \tau }(\delta + T(0))^2 (D+E) \int _{t-\tau }^{t} x_4^2(s) \mathrm{d}s. \end{aligned}$$

Then

$$\begin{aligned} \begin{aligned}&\mathrm {L}W(t,x_t) = \mathrm {L}W_1(t,x_t) + \mathrm {L}W_2(t,x_t) \\&\quad \le \left( \beta T(0) e^{-d_3 \tau } - 2d_1 \right) D x_1^2(t) \\&\qquad + \left( \left( \beta T(0) e^{-d_3 \tau } - 2(d_2 + k)\right) E + F k \right) x_2^2(t) \\&\qquad + \left( (k-2d_3) F + G p \right) x_3^2(t) \\&\qquad + \left( (p-2d_4) G + \beta T(0) e^{-d_3 \tau }(\delta \right. \\&\qquad \left. + T(0))^2 (D+E) \right) x_4^2(t). \end{aligned} \end{aligned}$$

Under conditions (9), we choose for sufficiently small enough $\delta > 0$,

$$\begin{aligned} \left\{ \begin{array}{cc} \begin{aligned} &{}D = \frac{-d_1}{\beta T(0) e^{-d_3 \tau } - 2d_1},\\ &{}E = \frac{-2 F k}{\beta T(0) e^{-d_3 \tau } - 2(d_2 + k)},\\ &{}F = \frac{-2 G p}{k - 2d_3},\\ &{}G = \frac{-\delta - \beta T(0) e^{-d_3 \tau }(\delta + T(0))^2 (D+E)}{p-2d_4}. \end{aligned} \end{array}\right. \end{aligned}$$

Then the disease-free equilibrium of (3) is stochastically stable. $\square$

### **Stability of the endemic equilibrium**

By centering the system (3) around $E^*$ using the transformations

11 $$\begin{aligned}&T = x_1 + T^*, \quad L = x_2 + L^*, \quad I = x_3 + I^*, \quad \text {and} \quad V = x_4 + V^*.\nonumber \\&\begin{aligned} dx_1(t)&= \left( d_1 T(0) - \beta e^{-d_{3} \tau } (x_1(t-\tau )+T^*) x_4(t-\tau )\right. \\&\qquad \left. - d_1 (x_1(t) + T^*) \right) \mathrm{d}t \\&- \sigma (x_1(t-\tau )+T^*) x_4(t-\tau ) dB(t), \\ dx_2(t)&= \left( \beta e^{-d_{3} \tau } (x_1(t-\tau )+T^*) x_4(t-\tau ) \right. \\&\qquad \left. - (d_2 + k) (x_2(t)+L^*)\right) \mathrm{d}t \\&+ \sigma (x_1(t-\tau )+T^*) x_4(t-\tau ) \mathrm{d}B(t), \\ dx_3(t)&= \left( k (x_2(t)+L^*) - d_3 (x_3(t)+I^*)\right) \mathrm{d}t, \\ dx_4(t)&= \left( p (x_3(t)+I^*) - d_4 (x_4(t)+V^*) \right) . \end{aligned} \end{aligned}$$

The corresponding linear system is

12 $$\begin{aligned} \begin{aligned} \mathrm{d}x_1(t)&= \left( - \beta e^{-d_{3} \tau } x_4(t-\tau ) - d_1 x_1(t) \right) \mathrm{d}t \\&\qquad - \sigma T^*x_4(t-\tau ) \mathrm{d}B(t), \\ \mathrm{d}x_2(t)&= \left( \beta e^{-d_{3} \tau } x_4(t-\tau ) - (d_2 + k) x_2(t) \right) \mathrm{d}t \\&\qquad + \sigma T^*x_4(t-\tau ) \mathrm{d}B(t), \\ \mathrm{d}x_3(t)&= \left( k x_2(t) - d_3 x_3(t) \right) \mathrm{d}t, \\ \mathrm{d}x_4(t)&= \left( p x_3(t) - d_4 x_4(t) \right) \mathrm{d}t. \end{aligned} \end{aligned}$$

#### Lemma 3.2

The zero solution of (12) is stable in mean-square if $R_0^s > 1$ and

13 $$\begin{aligned} \beta e^{-\mu d_3} T^*< \min \left\{ 2 d_1, 2(d_2 + k) \right\} , \quad k< 2 d_3, \quad p < 2d_4. \end{aligned}$$

#### Proof

Choose the Lyapunov functional

$$\begin{aligned}&W(t,x_t) = x_1^2(t) + Hx_2^2(t) + Kx_3^2(t) + Lx_4^2(t) \\&\qquad + \left( \sigma ^2 T^*+ \beta e^{-\mu d_3} \right) \left( H+1\right) T^*\int _{t-\tau }^{t} x_4^2(s) \mathrm{d}s, \end{aligned}$$

where *H*, *K*, *L* are arbitrary positive quantities to be determined. Then

$$\begin{aligned} \begin{aligned}&\mathrm {L}W(t,x_t) = 2x_1(t) \left( - \beta e^{-d_{3} \tau } x_4(t-\tau ) - d_1 x_1(t)\right) \\&\qquad + 2 H x_2(t) \left( \beta e^{-d_{3} \tau } x_4(t-\tau ) - (d_2 + k) x_2(t)\right) \\&\qquad + 2 K x_3(t) \left( k x_2(t) - d_3 x_3(t)\right) + 2 L x_4(t) \left( p x_3(t) \right. \\&\qquad \left. - d_4 x_4(t)\right) + \sigma ^2 (T^*)^2 x_4^2(t-\tau ) (H+1) \\&\qquad + \left( \sigma ^2 T^*+ \beta e^{-\mu d_3} \right) \left( H+1\right) T^*(x_4(t) - x_4^2(t-\tau ))\\&\quad \le \left( \beta T^*e^{-d_3 \tau } - 2d_1 \right) x_1^2(t) + \left( \left( \beta T^*e^{-d_3 \tau }\right. \right. \\&\qquad \left. \left. - 2(d_2 + k)\right) H + K k \right) x_2^2(t) \\&\qquad + \left( (k-2d_3)K + L p \right) x_3^2(t) + \left( \left( \sigma ^2 T^*+ \beta e^{-\mu d_3} \right) \right. \\&\qquad \left. \left( H+1\right) T^*+ (p-2d_4)L\right) x_4^2(t). \end{aligned} \end{aligned}$$

Under conditions (13), we choose

$$\begin{aligned} \left\{ \begin{array}{cc} \begin{aligned} H &{}= \frac{-2 K k}{\beta T^*e^{-d_3 \tau } - 2(d_2 + k)},\\ K &{}= \frac{-2 L p}{ k - 2d_3 },\\ L &{}= \frac{-2\left( \sigma ^2 T^*+ \beta e^{-\mu d_3} \right) \left( H+1\right) T^*}{p-2d_4}. \end{aligned} \end{array}\right. \end{aligned}$$

Consequently,

$$\begin{aligned} \begin{aligned}&{\mathbb {E}} \left[ \mathrm {L}W(t,x_t)\right] \le \left( \beta T^*e^{-d_3 \tau } - 2d_1 \right) {\mathbb {E}}|x_1(t)|^2 \\&\qquad - K k {\mathbb {E}}|x_2(t)|^2 - L p {\mathbb {E}}|x_3(t)|^2\\&\qquad -\left( \sigma ^2 T^*+ \beta e^{-\mu d_3} \right) \left( H+1\right) T^*{\mathbb {E}}|x_4(t)|^2\\&\quad \le - \min \left\{ 2d_1 - \beta T^*e^{-d_3 \tau }, K k, L p, \left( \sigma ^2 T^*\right. \right. \\&\qquad \left. \left. + \beta e^{-\mu d_3} \right) \left( H+1\right) T^*\right\} {\mathbb {E}}|x(t)|^2, \end{aligned} \end{aligned}$$

hence the zero solution of (12) is mean-square stable. $\square$

#### Theorem 3.2

Conditions (13) are sufficient for stability in probability of the endemic equilibrium $E^*$ of the nonlinear system (3) or the trivial equilibrium of (11).

#### Proof

Following the same argument of Theorem 3.1 by choosing

$$\begin{aligned} W(t,x_t)= & {} (M + N)T^*(x_1^2(t) + x_2^2(t)) + Q x_3^2(t) + P x_4^2(t) \\&+ \beta e^{-\mu d_3} T^*(\delta + T^*)^2 (M + N) \int _{t-\tau }^{t} x_4^2(s) \mathrm{d}s, \end{aligned}$$

where *M*, *N*, *Q*, *P* are positive quantities to be determined based on (13). $\square$

## Stability areas and numerical simulations

**Fig. 2:**
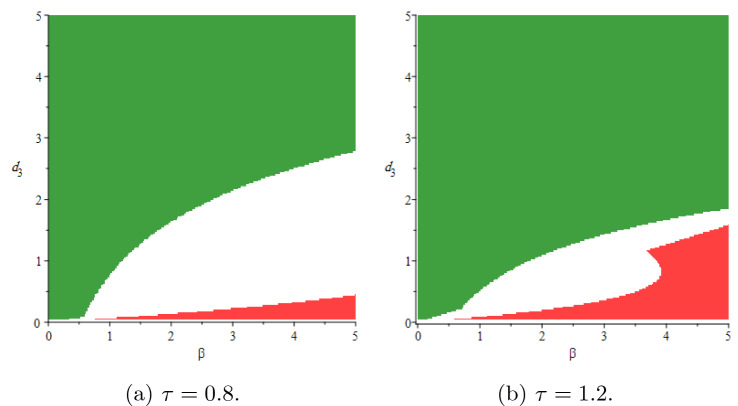
Stability areas of $E_0$ (green)(extinction) and $E^*$ (red)(persistence) (Colour figure online)

**Fig. 3:**
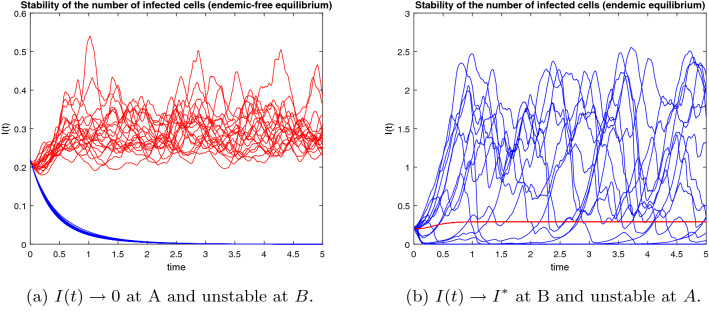
Numerical simulation of the solution of (3)

**Fig. 4:**
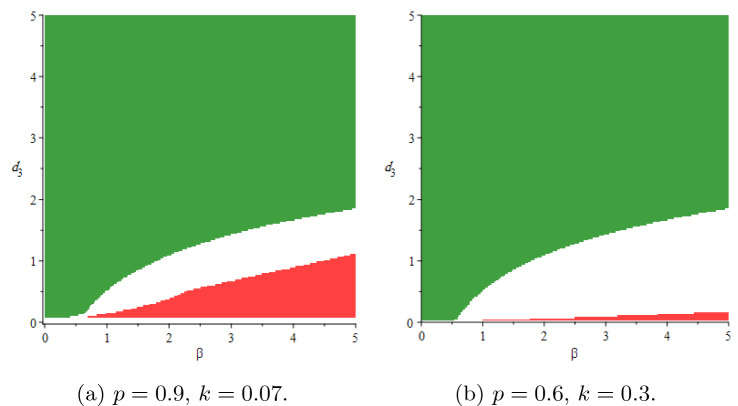
The effect of latent period *k* and the rate of virus particles *p*

**Fig. 5:**
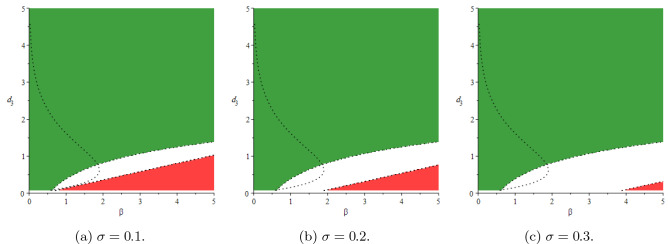
The effect of noise on the stability regions

In this section, we will show stability areas of the equilibrium states $E_0$ and $E^*$ in $(\beta ,d_3)$-space of parameters. Using the parameter values $T(0) = 5, \, d_1 = 0.9, \, d_2 = 1.1, \, p = 0.8, \, d_4 = 1.5, \, k = 0.05$ and based on conditions (9),(13), the stochastic stability regions of the disease-free equilibrium $E_0$ and the endemic equilibrium $E^*$ are shown in Fig 2 for different values of $\tau$. The delay $\tau$ has a reasonable effect on the stability regions, it increases the region of $E_0$. Consequently, it is advisable to increase the delay tactics which can be represented in the antiretroviral therapies, suitable licensed vaccine, etc. We perform the numerical simulation at specific points within the regions. At the point $A=(0.5,0.5)$ in the stability region of $E_0$, we simulate the number of infected cells in Fig 3a, we get 20 blue stable trajectories, and the number of infected cells goes to zero with $R_0^s = 0.0065 < 1$. In this figure, the equilibrium state $E_0$ is unstable at the point $B = (2.5,0.01)$ throughout simulating 20 red unstable trajectories. In Fig 3b, the endemic equilibrium $E^*$ is stable with $R_0^s = 1.6369 > 1$ at the point B, 20 red stable trajectories and the number of infected cells goes to the endemic equilibrium $I(t) \rightarrow I^*= 0.2894$. In the same figure, there are 20 blue unstable trajectories, i.e, the endemic equilibrium is unstable at the point *A*. We fix the delay and see the effect of the latent period *k* and the rate of free virus particles in Fig 4. If the latent cells take more time before joining the infected class of cells besides the decrease in the production rate of virus particles, this may help in eradicating the disease within-host. It should be noted the effect of the noise on the stability of the equilibrium states of (3). The noise parameter has a good effect on decreasing the stability region of the endemic equilibrium $E^*$ as shown in Fig 5.

**Fig. 6:**
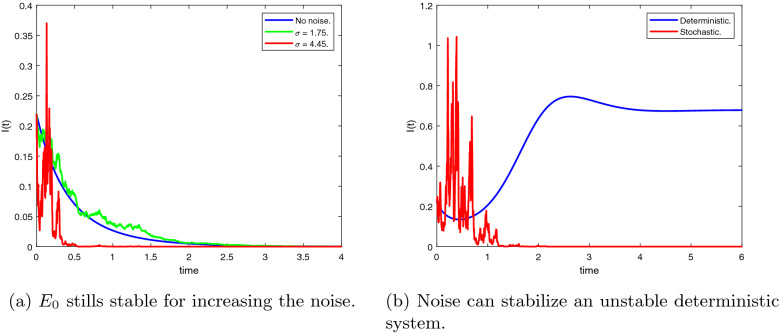
Solution behavior of deterministic and stochastic system for increasing noise

Moreover, the numerical simulations are performed to show the effect of the noise parameter throughout showing the behavior of the number of infected cells for different values of $\sigma$, see Fig 6a. The equilibrium state $E_0$ remains stable for increasing the noise parameter, and the number of infected cells goes to zero. Figure 6b compares the simulation of the solution of the deterministic system with the simulation of the stochastic system, and the noise can stabilize (red trajectory) an unstable endemic deterministic system (blue trajectory).

## Conclusion

This paper presents the extinction and persistence of the COVID-19 pandemic within-host through a stochastic mathematical model with time delay. Our results reveal that the delay tactics like antiretroviral therapies, suitable licensed vaccine and immune foods are very effective in eradicating the disease from the human body. Sufficient conditions for extinction and persistence of the disease within-host are obtained. One of the main results in this work is the importance of the noise effect in the mathematical model. Noise can stabilize an unstable pandemic deterministic system, and consequently, we can have the extinction via the stochastic model.

## Data Availability

The data sets generated and/or analyzed during the current study are available from the corresponding author on reasonable request.
